# Lack of reproducibility in osteocalcin-deficient mice

**DOI:** 10.1371/journal.pgen.1008939

**Published:** 2020-06-26

**Authors:** Takeshi Moriishi, Toshihisa Komori

**Affiliations:** 1 Department of Cell Biology, Nagasaki University Graduate School of Biomedical Sciences, Nagasaki, Japan; 2 Basic and Translational Research Center for Hard Tissue Disease, Nagasaki University Graduate School of Biomedical Sciences, Nagasaki, Japan; HudsonAlpha Institute for Biotechnology, UNITED STATES

Reproducibility of data and an author's responsibility to them are extremely important for science. Therefore, researchers repeat experiments and evaluate data objectively and confirm them with various methods. In the comments by Dr. Karsenty, he did not mention the bone phenotypes of osteocalcin-deficient (Ocn^–/–^) mice, published in *Nature* in 1996 [1]. That was the first paper describing Ocn^–/–^mice. Ocn^–/–^mice showed a drastic increase in both trabecular and cortical bone due to increased bone formation. Cortical thickness reached to 150% of wild-type mice. However, the bone volumes of both trabecular and cortical bone and bone formation in our Ocn^–/–^mice and Williams’s Ocn^–/–^mice were normal [2,3]. Furthermore, the bone phenotypes reported in Karsenty’s Ocn^–/–^mice were not reproduced in a recent paper that analyzed bone morphology and showed cortical thickness in the radius was normal [4].

There was another example of a lack of reproducibility in the transgenic mice generated by Karsenty’s group and our group. Transgenic mice expressing dominant-negative Runx2 (DNA binding domain only) by Karsenty’s group showed drastic reduction in both trabecular and cortical bone, bone formation was reduced to 30% of wild-type mice; the expression of osteocalcin, bone sialoprotein, and osteopontin was virtually abolished; and Col1a1 expression was markedly reduced [5]. These phenotypes were unexpected, because the expression level of dominant-negative Runx2 was much less than endogenous Runx2. The dominant-negative Runx2 inhibits Runx2 in a dose-dependent manner [6]. In our dominant-negative Runx2 transgenic mice, the level of transgene expression was more than ten times higher than endogenous Runx2, but the phenotypes were completely different from those in Karsenty’s group. We observed a mild increase in trabecular bone due to reduced bone resorption in our transgenic mice [6].

Since we were unable to reproduce the bone phenotypes of Ocn^–/–^mice, we examined bone quality, glucose metabolism, testosterone synthesis in testis, and muscle mass. Finally, we found a function for osteocalcin in bone. Osteocalcin directs the alignment of apatite crystallites parallel to collagen fibrils. However, we could not find any differences in glucose metabolism, testosterone synthesis in testis, or muscle mass between wild-type and Ocn^–/–^mice. To examine glucose metabolism, we measured body weights, blood glucose levels, HbA1c, and subcutaneous and visceral fat mass, and performed glucose tolerance test (GTT) and insulin tolerance test (ITT) in male and female mice at 11 weeks-18 months of age with normal or high-fat diet. However, all of the glucose metabolism phenotypes were normal in Ocn^–/–^mice. We performed GTT in 58 wild-type and 62 Ocn^–/–^mice, and ITT in 27 wild-type and 24 Ocn^–/–^mice with normal or high-fat diets. Even if Ocn^–/–^mice were fed a high-fat diet for three months, glucose metabolism was normal [2].

To examine testosterone synthesis in the testis, we measured testis weights, serum testosterone levels, the number of spermatozoa, the frequencies of abnormal spermatozoa, and germ cell apoptosis, and performed histological analysis of testis and epididymis and expression analysis of the genes, which are necessary for testosterone biosynthesis. All of the testosterone synthesis phenotypes were normal in Ocn^–/–^mice. In muscle, we examined the weights of quadriceps, gastrocnemius, soleus, and extensor digitorum longus, and the average area of muscle fibers. All of them were also normal in Ocn^–/–^mice. Testes, serum, and muscle were examined using the enough number of mice at different ages [2].

We did not perform osteocalcin injection into mice, because we pursued the physiological functions of osteocalcin rather than the biologic activity of the substance for a therapeutic use. The administration of osteocalcin was not in the scope of our work. Dr. Karsenty claimed that we cannot conclude that osteocalcin is not a hormone without the osteocalcin injection experiments. Treatment with vitamin K, which reduces uncarboxylated osteocalcin, does not affect glucose metabolism in humans, and warfarin, which increases uncarboxylated osteocalcin, has no effect on glucose metabolism in rats and there are no reported studies in humans [7,8]. Absence of osteocalcin has no effects on glucose metabolism, testosterone synthesis, and muscle mass in mice [2,3]. Moreover, the association of osteocalcin and improved glucose metabolism in humans can be explained by exercise-induced bone formation [2]. Thus, it is difficult to believe that uncarboxylated osteocalcin is a hormone. At the same time, we do not deny the biological activity of osteocalcin in multiple organs when it is administrated in a large amount. Dr. Karsenty mentioned GLP1 as a similar example of osteocalcin by saying that mice lacking GLP1R have a mild metabolic phenotype at best, but it plays critical roles in physiology. That is not true. GLP1R-decicient mice show apparent glucose intolerance [9]. Uncarboxylated osteocalcin is a biologically active protein, which exerts a hormone-like effect when administered.

Our Ocn^–/–^mice had been backcrossed with C57BL/6N more than 8 times. Karsenty's group analyzed Ocn^–/–^mice with mixed genetic backgrounds of C57BL/6J and 129/Sv. We chose to use a uniform rather than a mixed genetic background to reduce phenotypic variation, which makes it easier to detect mild abnormalities in our Ocn^–/–^mice. Furthermore, it has to be noted that C57BL/6J but not C57BL/6N mice have a mutation in the nicotinamide nucleotide transhydrogenase (*Nnt*) gene and show impaired glucose metabolism [10]. Mating between heterozygous mice always has to be done to get knockout and wild-type littermates for comparison. If mating between homozygous mice was performed, however, it is possible that introduction of the homozygous *Nnt* mutation into Ocn^–/–^mice but not into wild-type mice could have led to impaired glucose metabolism only in Ocn^–/–^mice. Even if this occurred, however, the unreproducible phenotypes in the testis and muscle still cannot be explained.
